# A Subtle Network Mediating Axon Guidance: Intrinsic Dynamic Structure of Growth Cone, Attractive and Repulsive Molecular Cues, and the Intermediate Role of Signaling Pathways

**DOI:** 10.1155/2019/1719829

**Published:** 2019-04-14

**Authors:** Xiyue Ye, Yan Qiu, Yuqing Gao, Dong Wan, Huifeng Zhu

**Affiliations:** ^1^College of Pharmaceutical Sciences and Traditional Chinese Medicine, Southwest University, Chongqing 400715, China; ^2^Chongqing Engineering Research Center for Pharmacological Evaluation, Chongqing 400715, China; ^3^Engineering Research Center for Chongqing Pharmaceutical Process and Quality Control, Chongqing 400715, China; ^4^Department of Emergency, The First Affiliated Hospital of Chongqing Medical University, Chongqing 400016, China

## Abstract

A fundamental feature of both early nervous system development and axon regeneration is the guidance of axonal projections to their targets in order to assemble neural circuits that control behavior. In the navigation process where the nerves grow toward their targets, the growth cones, which locate at the tips of axons, sense the environment surrounding them, including varies of attractive or repulsive molecular cues, then make directional decisions to adjust their navigation journey. The turning ability of a growth cone largely depends on its highly dynamic skeleton, where actin filaments and microtubules play a very important role in its motility. In this review, we summarize some possible mechanisms underlying growth cone motility, relevant molecular cues, and signaling pathways in axon guidance of previous studies and discuss some questions regarding directions for further studies.

## 1. Introduction

Proper axon guidance is essential in both the developing nervous system and the nerve regeneration process so as to insure integrity and precision of nervous system patterning. In the developing nervous system, axons project through considerable distance to their targets [1]. During embryonic development, each differentiating neuron sends out an axon, the growth cone is located at the tip of it, which senses the environmental change and leads the axon to migrate to its target. This pathfinding process is influenced by a combination of different aspects, for example, the motility of the growth cone, different guidance cues, and underlying signaling pathways. Evidences have been provided by experiments in both vertebrates and invertebrates [2, 3].

Not only that, axon guidance is also crucial in the nerve regeneration process. Nerve injury has always been one of the most common diseases that can be induced by crush, traction, ischemia, penetrating injury, etc. However, there exist some problems for the recovery after nerve injury, that is, poor regeneration and compromised functional recovery. Past researches had declared that the regeneration of the peripheral nerves is easier than the central nerves, or so to say, the central nerves can hardly regenerate because of a variety of reasons including inhibitory environment of the central nervous system (CNS) (for example, inhibitory molecules and formation of glial scars [4]). In spite of that, functional recovery needs proper axon navigation, which requires the axons to navigate along the path correctly and target the original position. The nerves would undergo several changes (for example, axon end “die back” and Wallerian degeneration) shortly after injury, and the initiation of a robust regeneration process can be observed after these changes, but they tend to fail as a result of inability to navigate in the proper direction [5]. During the navigation process, misdirection usually happens, which means that the motor nerves navigate incorrectly to the sensory nerves, or to the skin, and so for the sensory nerves [6]. For example, in the rat sciatic model, different injury model showed unexpected low accuracy of motor axon regeneration which led to disturbed functional recovery [7]. Obviously, misdirection places an obstacle for the functional recovery since that axons fail to reinnervate their original targets and result in substantial functional deficits. Hence, navigation of the axons needs to be extremely precise to guarantee the proper function of the targets in both the developing and regenerating nervous system.

For bilaterally symmetrical animals, integrated sensory inputs and coordinated motor control on both sides of the body are essential. Some neurons in the CNS project their axons to the opposite side of the body, whereas others project axons that remain on the same side. In insects, the so-called “midline cells” separate the two symmetrical halves of the CNS, while in vertebrates, midline cells form the “floor plate.” In the developing CNS, most of the central nervous system cells grow toward the midline at first while they primarily respond to attractive cues. And then the axons turn longitudinally with two patterns, either ipsilaterally (growing at their own side continuously) or contralaterally (crossing the midline and then turning anteriorly toward the brain) [8]. In vertebrates, the cell bodies of spinal commissural neurons differentiate in the dorsal spinal cord and project their axons ventromedially toward the floor plate in response to multiple cues. Attractive cues facilitate crossing the midline whereas repulsive cues cause the axons turn away from the midline. For example, netrin-1 could mediate midline crossing by attracting these axons. After midline crossing, commissural axons sort into distinct positions within the ventrolateral funiculus and are repelled by repulsive cues, like Slit, and never return the floor plate. Commissural axons are mainly sensitive to attractive cues before crossing. After crossing, they switch their responsiveness thus become insensitive to the attractive cues but sensitive to repulsive cues. This switch mechanism is getting more important in recent studies [9]. In the studies of the possible mechanisms of axon guidance, a large number of experiments were based on midline cells.

**Figure 1:**
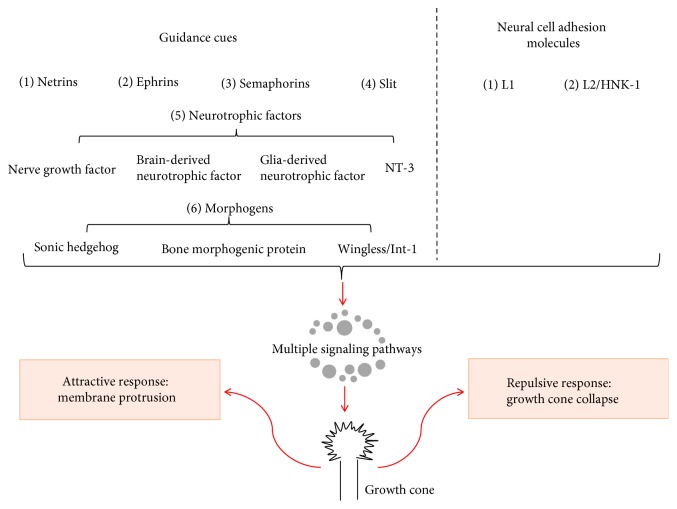
Multiple attractive and repulsive molecular cues, including guidance cues (netrins, ephrins, semaphorins, slit, neurotrophic factors, and morphogens.) and neural cell adhesion molecules (L1, L2/HNK-1), act on the growth cone and change the motility of it through multiple signaling pathways. The membrane protrusion of growth cone is responsible for attractive response towards molecular cues, while the collapse of growth cone is held accountable for repulsive response towards molecular cues.

In this review, we will first give an overview of the highly dynamic structure of growth cone, or in other words, the growth cone motility. Then, a summary of different repulsive and attractive molecular cues that mediate the growth cone guidance will be given. Lastly, we will present an outline of the signaling pathways that modulate the reactions of the growth cone toward different molecular cues (Figure 1).

## 2. Growth Cone Motility

Each axon is led by a highly motile structure, termed the “growth cone,” which is located at the tip of an axon. Growth cone samples different attractive and repulsive molecular cues and responds to them by modulating its dynamic structures, thereby guiding the axon to their target. The dynamic cytoskeleton of actin and microtubules in growth cone is fundamental for them to function well in axon guidance.

**Figure 2:**
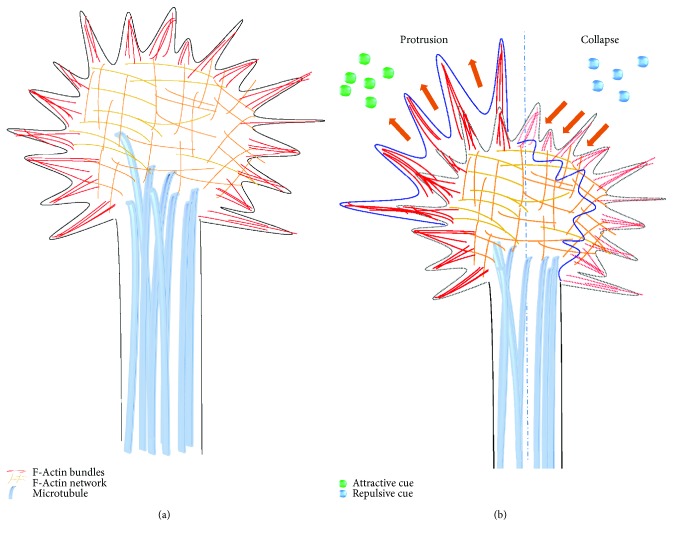
Schematic illustration of highly dynamic structure of a growth cone: dense microtubules located in the central domain that extend from axonal shaft to a growth cone to support growth cone movement and to serve as the tracks for transport of membranous organelles. In the peripheral domain, there are bundled actin filaments (F-actin bundles) retracting at similar rates at the leading edge and a network of short and branched actin filaments (F-actin network). Sometimes microtubules also explore the peripheral domain. (b) Schematic illustration of actin-based growth cone motility: attractive cues lead growth cone to protrude towards them. Protrusion is resulted from inhibited retrograded actin flow, where continued polymerization of actin at the leading edge, myosin motors, and microtubules rearrangement is involved. On the other hand, repulsive cues lead to actin filaments and microtubules dissolution and cause growth cone collapse that triggers the repulsive response towards the repulsive cues.

The sizes and shapes of the growth cones are divergent, ranging from a simple “paint brush” with a single tapering filopodium to a gigantic expansion bearing a rich efflorescence of filopodia and lamellipodia. The highly dynamic shapes of growth cones are related with several morphological distinct features on the surface that reflect the type of neurons to which the growth cones belong to, or the local environment of the growth cones, or their stage of development [10]. The basic structures of a growth cone can be divided into three parts based on cytoskeletal distribution: the central (C) domain, the peripheral (P) domain, and the transition (T) zone. The P-domain is primarily characterized by filopodia and lamellipodia. Filopodia are thin spike-like projections that consist of bundled actin filaments (F-actin bundles) which retract at similar rates. Along F-actin bundles, individual dynamic “pioneer” microtubules (MTs) may explore this area. Lamellipodia consist of a network of short and branched actin filaments (a mesh-like branched F-actin networks), which is punctuated by long cross-linked F-actin bundles [11]. Filopodia mainly function in sampling the extracellular environment, whereas lamellipodia mainly function in movement. In the cell migration process, filopodia were described as finger-like protrusions that continuously sense the environment [12]. The C-domain locates behind the P-domain. It is affluent with cellular organelles such as mitochondria and exocytotic vesicles. The pivotal characteristic of this region is the dense microtubule array, extending from the axonal shaft to the growth cone to support growth cone movement and to serve as the tracks for transport of membranous organelles. Between the P- and C-domains resides the T-region, where myosin contractile structures (termed “actin arcs”) regulate both actin and microtubules [13, 14]. In brief, the directional pathfinding process of an axon might result from either growth cone membrane protrusion towards attractive molecular cues or growth cone collapse induced by repulsive molecular cues. Membrane protrusion involves continued polymerization of actin at the leading edge, myosin motors, and microtubules rearrangement. On the other hand, collapse is accompanied by loss of F-actin from the leading edge of the growth cone and the subsequent loss of dynamic microtubules [15, 16] (Figure 2).

In order to understand the mechanisms of growth cone motility, we need to have a basic understanding of the basic structural units within the growth cone: actin. Actin is a highly conserved eukaryotic protein that forms microfilaments and is abundantly expressed in all tissues. It has three subtypes in mammalians: *α*, *β*, and *γ*. Under specific depolymerized condition, actin exists in the form of monomer (G-actin) with a single polypeptide of 42 kDa. While under polymerized condition, actin form helical filaments (F-actin), which undergo dynamic exchange process with small subunits, which contain different percentage of dimers and oligomers [17, 18]. Neuron cells mostly express *α* and *β* subtypes of actin [19]. The process of polymerization and depolymerization is pivotal for actin to exert its function. F-actin is considered to possess structural polarity with a “barbed” and a “pointed” end. Actin dynamic in cell is globally accepted that it bases on treadmilling, where actin filaments polymerize regularly at the barbed ends and depolymerize at the pointed ends [20].

**Figure 3:**
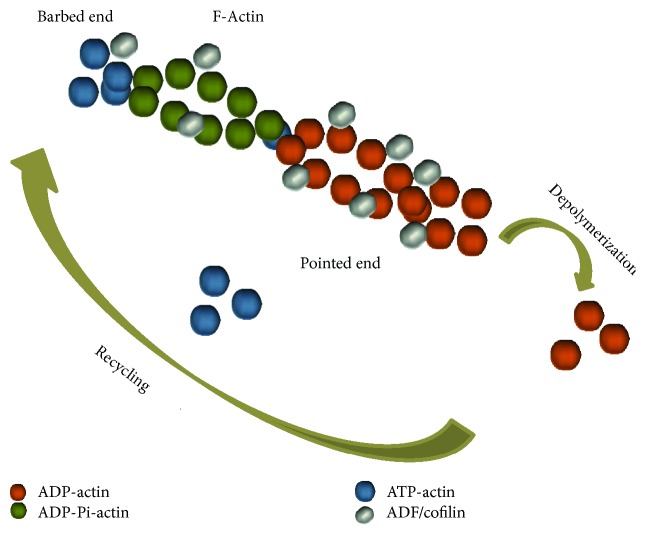
ADF/cofilin increases treadmilling of actin filaments. Treadmilling is fundamental for actin dynamic. In which process, actin filaments polymerize regularly at the barbed ends and depolymerize at the pointed ends. ADF/cofilin preferentially binds with the actin^ADP^ subunits in the F-actin and increases the rate of depolymerization from the pointed end. The different quantities of the different species drawn are meant to give an idea of their relative concentration at steady state.

In actin-based motile processes, actin-depolymerizing factor (ADF)/cofilin family of actin regulatory molecules are important. Early in the 1990s, the ADF/cofilin family was widely acknowledged to be responsible for the high turnover rates of actin filaments. It was proposed that ADF and cofilin cooperatively and preferentially bound the actin^ADP^ subunits in the F-actin, which would increase the rate of depolymerization from the pointed end, and kinetically limited the rate of barbed-end assembly, thus enhanced the directional shuttling of subunits through the filaments [21, 22] (Figure 3). So far, much has been learned about the participation of ADF/cofilin in axon guidance. The regulation process of many guidance cues on growth cone motility was elucidated to be tightly related with ADF/cofilin; the details will be discussed below in Attractive and Repulsive Molecular Cues anywhere related.

According to related experiments, the highly dynamic F-actin networks enable the shape change of growth cones. The motility of lamellipodia of most motile cells in P-domain is characterized by three steps: first, F-actin filaments assemble at the leading edge; second, F-actin filaments and bundles retrogradely flow from the leading edge (distal growth cone margin) to the central cytoplasmic domain, and probably driven by the action of myosin motors, and that the decreased rate of F-actin retrograde flow leads to the directional growth of the leading edge; and third, proximal F-actin recycle in the T-region maintains a steady state retrograde filament reflux [23]. Coupled with this, actin treadmilling was considered as the engine that generates the protrusive force in lamellipodia [12].

Previous experiment observed the retrograde flow of F-actin bundles in growth cone, where the process was sustained by ongoing actin polymerization at the leading edge and actin depolymerization at the proximal zone [24]. In the developing visual system in rat brain, retinal growth cones that were labeled fluorescently became more filopodial and larger as they traversed the optic chiasm, where they made a directional decision [25]. In the regeneration process, when growth cones were reorienting, actin accumulated in the P-domain and retrogradely flowed to C-domain along the filopodia [26]. Furthermore, it is suggested that the rate of retrograde F-actin flow was inversely proportional to the rate of C-domain extension. Taken together, these results suggested that the growth cone regulates the rate and direction of axons with participation of intracellular F-actin networks [27].

The role of myosin in retrograde F-actin flow was explored as well. In Medeiros et al.'s experiment, the function of myosin was blocked whether by gene inactivation or enzyme inhibition, both treatments led to dose-dependent attenuation of retrograde F-actin flow and filopodia growth, and the result suggested that the growth rate was directly proportional to myosin inhibition. It is proposed that F-actin retrograde flow resulted from two separated processes: actin assembly and myosin-based filaments retraction. This experiment was first to provide direct evidence for the involvement of myosin in retrograde F-actin flow [28]. Then more specific experiments were conducted trying to explain the function of myosin subtypes. To selectively block myosin II, blebbistatin was used, which was a specific myosin II ATPase that can make the bound between myosin II and F-actin weaker by trapping active myosin II in a certain state. It was found that myosin II inhibition led to an approximate 50% decrease in F-actin retrograde flow. After that, two possible forces were proposed to account for the possible force(s) that drived the remaining 50% retrograde flow in the presence of blebbistatin: the remaining myosin activity and/or the actin network at the leading edge. Trying to verify the proposal, cytochalasin B was used to shut off barbed-end actin assembly at the leading edge both with and without the presence of blebbistatin, respectively. Result showed that adding cytochalasin B alone had no acute effect on F-actin retrograde flow. In contrast, when growth cones were pretreated with blebbistatin and then treated with blebbistatin plus cytochalasin B, residual actin network was strongly inhibited. After blebbistatin was washed out while cytochalasin B was still in the presence, rapid clearance of F-actin retrograde flow was observed. Together, these results suggested that myosin II contractility and actin assembly accounted for a large percentage of F-actin retrograde flow, and with the absence of myosin II contractility, actin assembly could drive F-actin retrograde flow about 50% of the control rate [29]. On the other hand, there is another experiment demonstrating that myosin II was tightly related with actin filaments turnover in growth cone [29].

In spite of the actin cytoskeleton, microtubule is another instructive part in directional steering of the growth cones. Microtubules can initiate actin skeleton, and the directional movement of the growth cones is modulated by the interplay of these two cytoskeletal systems [30]. It is said that, during the process of a growth cone to avoid an inhibitory guidance cue, the microtubules would rearrange to accomplish turning, and F-actin is required for microtubule reorientation [31]. In Schaefer et al.'s experiment, multimode fluorescent speckle microscopy and correlative differential interference contrast imaging were used to investigate the interaction between actin bundles and microtubules. Result showed that filopodia and actin arcs interacted with microtubules strongly. And retrograde microtubule transport was observed that microtubules in P-domain flow retrogradely at the same rate as surrounding F-actin 65% of the time, suggesting a second function of filopodia in clearance of microtubule from P-domain [32]. However, less has been learned about the mechanisms or molecules that participate in the interplay between these two cytoskeletal systems. Latest research proposed that microtubule plus-end tracking proteins (+TIPs) might link the two cytoskeletons together and that +TIPs coupled microtubules to actin filaments [33].

Other studies also proposed that the motility of growth cone might be related with *β*-actin mRNA and its zipcode-binding protein and possibly mediate the attractive effect of certain guidance cue [34–36]. The mitochondrial dynamics also regulate growth cone guidance in retinal ganglia cells (RGCs), and this mechanism was reviewed a few years ago [37, 38].

## 3. Attractive and Repulsive Molecular Cues

In this section, we summarize some molecular cues that act on the growth cone, probably via regulating growth motility and subsequently influence the directional decision making of the growth cone. It is worthwhile mentioning that these molecular cues are not categorized by their attractive or repulsive effect on the growth cone because of intricate situations. *In vivo*, growth cones simultaneously encounter attractive and repulsive guidance cues, and thus, the behaviors of growth cone during axonal pathfinding reflect the complex integration of multiple signaling activities. For example, one molecular cue may be regarded as an attractant of a growth cone in most of the (similar) experiment conditions. However, with the presence of other factors, the same molecular cue can trigger repulsive turning response of the same growth cone. Therefore, in this section, we do not give absolute definition to the molecular cues. In other words, instead of characterizing them in terms of the attractive or repulsive turning response they can trigger of the growth cone, we review the possible effects they may have on the growth cone in different experiment conditions: in the CNS or the peripheral nervous system, in the developing nervous system or the regenerating process, in different species, etc., and give examples when the effect of a guidance cue may change.

### 3.1. Neural Cell Adhesion Molecules (NCAMs)

Cell adhesion molecules (CAMs) are ligands that participate in the cell-cell recognition during the tissue formation period. The better characterized CAMs belong to immunoglobulin (Ig) superfamily, comprised of neural cell adhesion molecules (NCAMs) and L1 with a shared L2/HNK-1 carbohydrate epitope.

NCAMs are cell surface glycoproteins, distributing along every area of differentiated nerve cells [39, 40]. Early in the 1980s, NCAMs were discovered to have a function in cell-cell interactions with the participation of some other pleiotropic effects [41]. Researches had been done in the chicken optical system. Antibody against NCAMs was used. It is suggested that suppression of the NCAM-mediated adhesion pathway resulted in a distortion of optical pathway [42]. In mice and flies, NCAMs mediated interaxonal adhesion, allowing growth cones to leave bundles and explore new paths [43]. Other researches related NCAMs to polysialic acid (PSA), which is a glycosylation on the surface of NCAMs. Basically, the role of PSA was thought to mediate cell-cell interactions and to create plasticity of cells [44, 45]. Experiment on chicken eyes suggested the involvement of PSA on NCAM to regulate the pathfinding process of retina ganglion cell axons in the developing nervous system [46]. What other experiments found was consistent with this experiment. Experiment on chick embryos suggested that PSA attenuated axon-axon interactions in the plexus and allowed axon reorganization, which is essential for specific motor neuron projection. Removal of PSA caused motor neuron guidance error which suggested that PSA was critical in specific motor neuron pathfinding [47, 48]. However, further study proposed that PSA only facilitated axons to respond to other guidance cues but did not affect the pathfinding process directly [49].

#### 3.1.1. L1

L1 subfamily of Ig superfamily serves as hemophilic and heterophilic receptors for lots of cell surface ligands. Expression deficiency of L1 resulted in corticospinal axon guidance errors, suggesting the function of L1 in the CNS axon guidance [50]. L1 also function together with other axon guidance cues to regulate axon guidance response, such as semaphorin3A [51, 52]. Latest experiment revealed the function of close homolog of L1 (CHL1, member of mammalian L1 family) in axon guidance. CHL1 deletion in mice caused a mistake in somatosensory thalamic axon projections, also interfered with semaphorin3A expression [53]. Then, the enzyme that mediated CHL1 function was suggested to play a role in axon guidance as well: the integrated function of BACE1 (the *β*-secretase enzyme that initiates production of the *β*-amyloid peptide involved in Alzheimer disease) was necessary for CHL1 to exert the function in axon guidance in the hippocampus [54].

The role of L1 in the regeneration of the peripheral nerve was explored. Experiment in a femoral nerve section model in rats trying to analyze the expression changes at the proximal and distal ends of nerves at various time points after injury provided a foundation for investigating the L1 effect on peripheral nerve chemotaxis regeneration. For example, it is suggested that L1 expression was higher in the sensory nerves than in the motor nerves at 2 weeks after injury; L1 expression was higher in the motor nerves than in the sensory nerves at the proximal end after injury, but its expression was greater in the sensory nerves at 2 weeks, suggesting that the second week might be a key period of chemotaxis regeneration. Meanwhile, in consistency with their previous study that L1 expression in the sensory nerves of normal rats was 5.8 times than that of the motor nerve, the high expression of L1 at the proximal end of the sensory nerves suggested that L1 is closely linked to chemotaxic regeneration [55].

#### 3.1.2. L2/HNK-1

L2/HNK-1 are carbohydrate epitope shared by several neural adhesion molecules. They are carried by several neural recognition molecules and were implicated to be important in cell-to-cell and cell-to-laminin adhesion [56].

At around 1990, preferential expression of the carbohydrate epitope L2 by Schwann cells was identified. In the regeneration stage of axons, higher L2 expression level was detected at the original target and much lower level in the inappropriate target. Through indirect immunofluorescence on fresh frozen sections, ventral roots of adult mice were found to express the L2 carbohydrate by myelinating Schwann cells, whereas few myelinating Schwann cells of the dorsal spinal roots expressed this carbohydrate. To further explore the impact of the preferential expression pattern of L2 carbohydrate, L2 antibodies were used to block its function. As a result, reduced neurite outgrowth was observed on motor neurons on ventral roots but not on dorsal roots. This observation suggested that L2 carbohydrate promoted neurite outgrowth of motor neurons and might thus contribute to the preferential motor neuron regeneration, or so to say, contribute to the pathway-selective reinnervation of motor nerves, which is another important phenomenon in nervous system patterning with unclear mechanisms [57, 58]. Regarding the role of HNK-1, a 3′-sulfated glucuronic acid, presenting on membrane-bound cell recognition molecules, experiments declared its participation in axon guidance. Its antibody was injected to zebrafish embryos; axon misrouting was observed [59]. Similarly, the role of HNK-1 was explored in regenerating retinotectal projection in goldfish; result suggested the role of HNK-1 in mediating retinal axon guidance [60]. A recent study on HNK-1 after SCI in adult zebrafish suggested that HNK-1 expression was upregulated in only those neurons that were intrinsically capable of regeneration and contributed to functional recovery after SCI, which might imply a role of HNK-1 in axon regeneration but the extent of which contributes to directional regeneration remains to be demonstrated [61].

Only recently has attention been directed to guidance cues and the signaling pathways; few researches are focusing on cell adhesion molecules.

### 3.2. Guidance Cues

Guidance cues were described dating back to 1960s and were regarded as either (1) broad gradients that were longitude and latitude markers that could be utilized by the axons to orient towards the targets, and the orientation was established by chemical gradients of specific chemical agents, or (2) substrate pathways, which were preexisting substrate routes, or a set of aligned guidance cues. The axons were guided along these routes in the developing nervous system [62–64].

The well-known guidance cues include neurotrophic factors, netrins, semaphorins, ephrins, slit, and the nonconventional morphogens, including Wingless/Int-1 (Wnt), sonic hedgehog (Shh), and bone morphogenetic protein (BMP) families.

#### 3.2.1. Neurotrophic Factors

Neurotrophic factors act as target-derived trophic factors and have a broad spectrum of biological functions in several tissues, including promoting neuronal survival and neurite outgrowth. Among all the neurotrophic factors, some play a role in mediating axon guidance, including nerve growth factor (NGF), brain-derived neurotrophic factor (BDNF), glia-derived neurotrophic factor (GDNF), and neurotrophin-3 (NT-3). These neurotrophic factors exert different effects on growth cones. They bind to various tyrosine kinase receptors (TrkA, TrkB, and TrkC) with high affinity to mediate growth turning, as well as bind to the neurotrophin receptor p75 (p75^NTR^) with low affinity to mediate filopodial dynamics and subsequently mediate growth cone motility [65–67]. Each Trk receptor demonstrates chemical affinity to specific neurotrophic factors. TrkA preferentially binds NGF, TrkB binds BDNF and NT-4/5, and TrkC binds NT-3. Despite the fact that neurotrophic factors are normally regarded as chemoattractant on growth cones, which means that they usually cause an attractive turning response of a growth cone towards them, it is worthwhile being aware that different neurotrophins may possess chemoattractive or chemorepulsive effects on the same growth cone under different conditions [68].

*(1) Nerve Growth Factor (NGF)*. Nerve growth factor is the first identified neurotrophin. The chemotropic effect was first studied on the sensory neurons and sympathetic neurons in the 1960s [68]. Experiments suggested that NGF was not accountable for long-range axon guidance; instead, it mediated local attractive response through TrkA receptor [65, 69, 70]. The process that the growth cone turns toward NGF was implicated to be mediated by activation of ADF/cofilin that promoted actin polymerization and subsequently growth cone turning towards NGF gradient [71]. In previous studies, with a biased turning model, where soluble NGF gradients helped to determine the angle that neurites turned, NGF induced chemoattractive response, although absolute control was not achieved as neurites still grew to channels without NGF gradients [72, 73]. However, in the nervous system, guidance cues do not work alone, thus the interplay among these proteins is necessary to analyze. Leipzig et al. was the first to study the response of coimmobilize biotinylated NGF (bNGF) and biotinylated Sema3A (bSema3A) in a single region at varying concentrations in dorsal root ganglia. It was indicated that axon responsiveness to a multicued coimmobilized model was sensitive and complex, and their strategy might be applied to future direct application in the nervous system injury models [74]. Recently, the combination of the experimental and computational model has been exploited to mimic axon pathfinding process, a significant chemoattractive response toward the NGF gradient was observed, while some neurites were still found in the end with no NGF gradient [73]. These studies demonstrated the complexity in axon guidance that single factor was not enough to support the whole process but with multiple guidance cues and other factors.

NGF also has a potential role in promoting axon regeneration and functional recovery in adult after injury [75, 76]. Because of the poor physiochemical stability and low ability to cross the blood spinal cord barrier of NGF, heparin-poloxamer (HP) hydrogel was constructed to wrap NGF and investigate the role of it in SCI. Result suggested that the locomotor function was gradually restored with treatment of NGF, while the NGF-HP hydrogel group showed the most significantly improved locomotor function recovery [77]. Another study found that intranasal NGF not only promoted axon regeneration but also improved locomotor behavior in rats with SCI [78]. Despite the improved regeneration and functional recovery, the exact role of NGF in guiding regrowing axons after injury needs to be further elucidated.

*(2) Brain-Derived Neurotrophic Factor (BDNF)*. BDNF was initially regarded as an essential factor for sensory neuronal survival in establishing or maintaining innervation [79]. Later, it was elucidated that BDNF has a wide range of functions in the nervous system, varying from promoting neural survival and differentiation, participating in the formation of appropriate synaptic connections in the brain to mediating growth cone guidance [80]. Normally, a gradient of BDNF was believed to trigger an attractive turning response of the growth cone. In the first place, BDNF is capable of mediating growth cone motility. It was suggested that BDNF stimulated filopodial number and length on growth cones of chick embryo retinal ganglion cell and dorsal root ganglion axons by increasing filopodial protrusion rates. Among which, retinal growth cone motility was regulated by BDNF through the activation of ADF/cofilin, and this mechanism was independent of myosin II activity which also enabled to increase filopodial length [81]. Coupled with these studies, its chemoattractive turning effect on a growth cone was suggested to need the participation of Ca^2+^ and the activation of transient receptor potential canonical channels (TRPC) [82].

BDNF is also one of the best characterized neurotrophic factors promoting axon regeneration and functional recovery [83–85]. After SCI in the rat, gene-modified human bone marrow-derived mesenchymal stem cells that continuously secreted BDNF were transplanted into the acute SCI model. Result indicated that locomotor recovery was improved, and BDNF might be associated with improved functional outcome in acute SCI. However, another study which also investigated functional outcome after transplantation of biomaterial that could express BDNF in the SCI model did not observe improved functional outcome. The difference between these studies is that the lesion sites of the spinal cord were different so that the requirement of the extent of precise retargeting might be different [86]. Functional outcomes influenced by BDNF were explored frequently in the cases of combined treatment. For example, it has been shown that overexpression of BDNF in the SCI model could trigger spasticity-like symptoms [87]. Considering the complexity of combined treatment, it is important to explore the mere role of BDNF in axon regeneration.

As indicated previously, the attractive or repulsive effect of the same guidance cue on the same growth cone may be inversed with the presence of other factors. According to Song et al., a gradient of BDNF induced repulsive turning of growth cones in the presence of a competitive analogue of cAMP or of a specific inhibitor of protein kinase A [88].

*(3) Glia-Derived Neurotrophic Factor (GDNF)*. GDNF acts as chemoattractant for various neuronal projections. It was believed that GDNF mediates these effects via two main signaling receptors: Ret (a transmembrane tyrosine kinase receptor) and NCAM, both of which requires GFRa1 as a receptor for proper ligand binding and activation. The intersections between GDNF and other guidance cues were implicated as well [89]. The positive regulation of GDNF in mediating the repulsive Seme3B signaling required NCAM but not Ret in commissural axon guidance [90]. GDNF also has a crosslink with ephrins. Latest study proposed that reverse signaling of ephrin-As was mediated by Ret that transmitted GDNF signaling upon interaction with the ligand-binding glycosylphosphatidylinositol (GPI) receptor GFRa1 [91]. In the peripheral nerve injury model, spatially and temporally controlled released GDNF enhanced axon regeneration and functional muscle reinnervation. In the context of chronic denervation, GDNF levels would continuously go down and place an obstacle for regeneration. Hence, the strategy that can control the spatial and temporal delivery of GDNF might be used to promote axon regeneration after peripheral nerve injury [92].

*(4) NT-3*. In the developing model, locally applied NT-3 attracted the developing corticospinal tracts (CST) in rats. To be specific, NT-3 directed the growth of the CST collateral branches from the white matter tract into the spinal gray matter target areas. In the injured model, lesioned adult rat corticospinal fibers regrew toward locally applied NT-3 while collagen was used as a vesicle. Also, in the injured adult CNS, increased NT-3 expression in the correct target significantly promoted regeneration into the appropriate region [93]. Taken together, these studies suggested that NT-3 may possess the attractive effect towards the axons whether in the developing nervous system or nerve regeneration after injury [94]. Latest study also suggested that during the process of the cephalic neural crest cells (NCCs) invading the optic vesicle region in chick embryos, NT-3 was involved in this chemotactic guidance of NCCs [95].

#### 3.2.2. Netrins (UNC-6)

Netrins are large (~70–80 kDa) soluble proteins with amino acid sequences that are similar with proteins of the laminin family. They can function as diffusible attractants and repellants in different situations.

Two kinds of netrins were purified from embryonic chick brain, which were netrin-1 and netrin-2. In chickens, netrin-1 is expressed by floor plate cells, while netrin-2 is expressed in the ventral two thirds of the spinal cord. Both netrin-1 and netrin-2 were believed to serve as chemoattractant at the beginning [96, 97]. Netrins were suggested to be important in commissural growth cone guidance and were expressed throughout embryogenesis [98]. However, later research in *Drosophila* indicated the bifunction role of netrin-1, where it was a chemoattractant for ventrally directed commissural axons whereas it was a chemorepellent for trochlear motor axons [99]. Another experiment *in vitro* showed that the repellent function of netrin-1 was depended on the status of cytosolic cAMP-dependent activity [100]. Experiment had also been done on rodent. A complete netrin-1 null animal showed much more severe axon guidance defects than netrin-1 loss-of-function gene-trap mice, indicating the importance of netrin-1 in axon guidance in the vertebrate [101].

The receptors of netrins were identified then: Deleted in colorectal cancer (DCC) and UNC-5. One of the homologs of DCC is UNC-40, which primarily affects ventral migration [102, 103]. DCC, a transmembrane protein of the Ig family, expressing on spinal commissural axons, was suggested to possess netrin-1-binding activity as a receptor of netrins. Netrin/DCC exerted the guidance function on retinal axons in *Xenopus*, vagal sensory axons in rodent, and olfactory sensory axons in zebrafish [104–106]. At that time, whether DCC could function alone to mediate the response of netrins was not clear [107, 108]. Then a following study, using hermaphrodite distal tip cells of *C. elegans* as a model, suggested that DCC and UNC-40 could mediate axon guidance independently and cooperatively. [109]. Latest research indicated that Down's syndrome cell adhesion molecule (DSCAM), expressing on spinal commissural axons, possessed binding affinity to netrin-1, collaborating with DCC and mediating commissural axon guidance in vertebrates [110, 111]. Another receptor for netrins is UNC-5. In rat, two homologs of UNC-5 were identified: UNC5H1A and UNC5H2 (both have immunoglobulin-like domains like UNC-5). Both were believed to be receptors of netrins as well. Normally, it is proposed that the DCC family mediated axon attraction response while the UNC-5 family mediated repulsion response toward the growth cone. However, DCC-mediated attraction was suggested to be able to convert to UNC-5/DCC-mediated repulsion by forming a receptor complex of UNC-5/DCC, which was triggered by netrin-1, while spontaneous suppression of the interaction between their cytoplasmic domains might be the underlying reason [112, 113].

Then the mechanisms of UNC-40-mediated response were explored. UNC-40 is regulated by UNC-73 and kinesin-related VAB-8 protein at its upstream. MIG-2 GTPase (one of the genetically identified targets of UNC-73) was activated at first, and then they affected the subcellular localization of UNC-40 [114]. Random fluctuation of UNC-40 activity of a neuron was suggested to affect this process. The response of UNC-40 to netrin was described as a stochastic process that evolved in time via random change [115]. Latest research indicated that the gene *madd-2* could promote the attractive response attributed to netrins and that the MADD-2 (proteins encoded by gene *madd-2*, related with the human developmental disorder Opitz syndrome) could potentiate the UNC-40 activity [116]. Netrins can change the polarity of growth cone protrusions in *C. elegans*, or so to say, the balance between UNC-40 driving protrusion and UNC-5/UNC-40 inhibiting protrusion [117, 118].

Another possible receptor of netrin has been proposed recently. The structures of a functional netrin-1 region were determined and provided evidence for neogenin to be a receptor of netrin-1. Neogenin was similar with DCC in structure and could bind with netrin-1 and netrin-3. Based on the determined structure, the researchers proposed that netrin created ligand/receptor signaling, assembled at the neuronal surface by binding to and bringing together receptor molecules via its two binding sites [119].

The effect of netrin-1 in peripheral nerve transection injury is also explored. In the nerve transection model, uninnervated Schwann cells would shed their myelin and proliferate to form Büngner bands together with other cell types. Büngner band could serve as a bridge in the nerve gap and direct the axons to navigate to their original targets again. The regeneration process often occurs within Büngner bands. Experiments suggested that netrin-1 was expressed in the Schwann cells of the intact peripheral nerves and was upregulated in Schwann cells of the distal nerve segment after peripheral nerve transection injury. With respect to that, different receptors of netrin-1 have different expression patterns in the intact peripheral nerves and injured nerves. *In vitro* experiments indicated that netrin-1 could promote proliferation and migration of Schwann cell through Unc5b receptor. Moreover, netrin-1 promoted Schwann cell migration was shown to be mediated by the p38 MAPK and PI3K-Akt signal pathway [120]. Since Schwann cell proliferation and migration is important in guiding the regenerating axons grow through the nerve gap, these results might relate netrin-1 to proper peripheral axon guidance after injury, and the netrin-1/Unc5b system is likely to serve as a new therapeutic strategy for PNS regeneration [121, 122].

It seems that, netrins, instead of being a conventional guidance cue merely, it might link neuron survival and guidance functionally [123]. However, there emerged some new opinions on the role of netrin-1 that were opposed to that in the previous studies. Ntn1 conditional knockout mouse line was used where netrin-1·expression in floor plate cells was selectively ablated. Results showed that in the absence of floor plate-derived netrin-1, the hindbrain and spinal cord commissural axons developed normally. Furthermore, with high expression of netrin-1 in the ventricular zone, Ntn1 deletion from the ventricular zone had the same commissural axon guidance defects as previously studied Ntn1-knockout mice. These findings showed that the previous view was inaccurate about the attraction response of commissural axons mediating by a gradient of floor-plate-derived netrin-1, but that netrin-1 primarily acted locally by promoting growth cone adhesion [124]. And that it is netrin-1 supplied by neural progenitors, not floor plate cells, that guided commissural axons in the developing spinal cord; the deposition of netrin-1 on the pial surface was a growth substrate that directed ventral axon guidance [125].

Some relevant signaling pathways were explored then. The c-Jun N-terminal kinase 1 (JNK1) pathway, one of the subfamilies of the MAPKs, is essential in brain development and strongly expressed in the nervous system. Both *in vitro* and *in vivo* experiments showed that inhibition of JNK-1 inhibited axon attraction mediated by netrin-1 in the presence of DCC or DSCAM. The result suggested that JNK1 was important in netrin/DCC and netrin/DSCAM signaling in the developing nervous system [126]. Furthermore, TUBB3, the most dynamic b-tubulin isoform in neurons, was proposed to directly interact with DCC and was important in netrin-1-mediated microtubule dynamics in guiding commissural axons *in vivo* [127].

#### 3.2.3. Semaphorins

Semaphorins are defined by a conserved ∼500 amino acid extracellular Sema domain, comprising a large family of secreted and transmembrane proteins, some of which function in axon guidance [128, 129]. Semaphorins are classified into at least eight classes in terms of the domain organization of their primary structure and the species origins. The receptors of semaphorins fall into two big families, the plexins and neuropilins (NRP). Four members of the plexin family (plexin-As; plexin-A1, -A2, -A3, and -A4) and two members of the neuropilin family (neuropilin-1 and neuropilin-2) were proved to be receptors for class 3-secreted semaphorins and being potent neural chemorepellent. Among the large family of semaphorins, semaphorin 3A (Sema3A) was studied in the greatest details, which is a member of class 3 semaphorin [130]. Plexins and neuropilins were suggested to form complexes to mediate axon guidance by Sema3A [131–133]. Sema3A mediated the repellent response by inducing growth cone collapse. Collapsin response mediator protein-2 (CRMP2) was identified as an intracellular protein mediating Seme3A-induced growth cone collapse [134]. Recently, Sema3A-mediated growth cone collapse has been suggested to have connection with a major drug target of Alzheimer's disease: BACE1 (beta-site amyloid precursor protein-cleaving enzyme 1). CHL1 fragment was generated by BACE1 upon Sema3A binding, and the fragment relayed Sema3A signal via ezrin-radixin-moesin (ERM) proteins to the neuronal cytoskeleton. That is to say, CHL1 and BACE1 controlled axon guidance by regulating growth cone dynamics [135]. The Rab family of small monomeric G proteins was suggested to involve in this process as well. Activation of Rab5 mediated Sema3A-mediated growth cone collapse in the developing nervous system in rodent [136].

Plexins were identified first as antigens for the monoclonal antibody MAbB2, expressing in the optic tectum in *Xenopus* tadpoles [137]. cDNA cloning and sequencing of *Xenopus* plexins revealed that they mediated homophilic cell adhesion [138]. Genetic and biochemical analysis in *Drosophila* suggested that repulsive response was mediated by plexin B (plexin B is endogenously expressed by the CNS neurons) with the participation of the Rho family GTPases. Both plexin B and P-21-activated kinase (PAK) are downstream effectors of active Rac; they competed with each other to bind with active Rac in the same GTP-dependent manner. PAK mediated the major signaling output of Rac to actin polymerization, which was essential for growth cone to turn. The binding of plexin B with Rac downregulated the PAK output with the participation of seven amino acid sequences in the cytoplasmic domain. Meanwhile, plexin B also possessed binding affinity to RhoA to upregulate its output, whereas the binding mechanism of plexin B with RhoA has been unknown yet [139]. On the other hand, NRP serve as receptors of semaphorins as well, while NPR-1 and NPR-2 bind differently with different class three semaphorins [140, 141]. In rodent, NRP-1 was thought to allow endothelial tip cell filopodia to protrude in a new direction at a specific location in the developing CNS [142]. NPR-2 was indicated to regulate a central projection of sensory axons in the spinal cord and the anterior commissure [143]. Except acting alone, NRP-1 and NRP-2 could also cooperate to mediate guidance of cranial neural crest cells and position sensory neurons via forming complexes with semaphorins [144]. Latest research proposed that semaphorin 6B (Sema6B) bound to floor plate-derived plexin A2 for navigation at the midline where a *cis*-interaction happened [145].

More recent study showed that other than acting as ligand to activate plexins or NRP, Sema-1a could also signal in reverse as a receptor [146]. Individual research demonstrated that Sema-1a reverse signaling played an important role in mediating midline crossing. Typically, Sema/plexin signaling is associated with repulsive response in terms of inhibiting midline crossing. However, according to Hernandez-Fleming et al., in the *Drosophila* CNS, Sema-1a functioned as a receptor to promote midline crossing, thus resulting in an attractive response instead of the typical repulsive response. Furthermore, Sema-1a-promoted midline crossing is independent of its canonical binding partner plexin A. Instead, the secreted Sema-2s functioned as attractive or adhesive ligands for Sema-1a-mediated midline crossing [147].

#### 3.2.4. Ephrins

Ephrins can be grouped into two types: ephrin-As and ephrin-Bs. A glycosylphosphatidylinositol (GPI) anchors ephrin-As to the cell membrane, and ephrin-Bs contain a transmembrane domain followed by a short cytoplasmic domain. The receptors of ephrins belong to the Eph family, the largest subgroup of receptor tyrosine kinases. Ephrins mediated both attractive and repulsive responses toward a growth cone.

In terms of the mechanisms of ephrin-As and ephrin-Bs, respectively, a possible mechanism in regulating ephrin-As activity is reverse signaling that triggers attractive and repulsive response relying on different coreceptors. p75 neurotrophin receptor was suggested to be involved in this reverse signaling in axon repulsion in developing retinal axons in mice. And genetic evidence was found in motor axons as well [148, 149]. The mechanisms of ephrin-Bs in mediating axon pathfinding overlap some of ephrin-As. Continuously, analysis of mutations in B-type ephrins revealed the role of reverse signaling in ephrin-Bs-mediated axon guidance, including ephrin-B1 and ephrin-B2 [150–152], and this reverse signaling was possibly regulated by the Src family kinases (SFKs), which was known as positive regulators of phosphotyrosine-mediated reverse signaling [153].

The receptors of ephrins are Eph receptors, members of tyrosine kinases receptors. Eph receptors are divided into two types (EphA and EphB) in terms of amino acid sequence and ligand specificity. EphA contains eight subtypes: EphA1-EphA8, while EphB contains five types (EphB1–EphB4, EphB6) [154, 155]. EphA receptors played a role in commissural axon guidance in chicken hindbrain and topographic mapping in mouse corticothalamic projections [156, 157]. EphB receptors were important in regulating repulsive response towards axons in the ventral midline and in the retinal system in the developing nervous system of mice [158–160]. Functions and possible mechanisms of relevant subtypes were explored. Ephrin-A4 mutant mice displayed aberrant midline axon guidance and defective spinal cord central pattern generator activity, mediating Rac-specific GTPase-activating protein *α*2-chimaerin [161]. In the recent years, researchers found that mice with genetically abolished EphA4 cleavage had motor axon guidance defects. EphA4 cleavage was proposed to be important to establish the concentration differential of active ephrins [162]. The signal transduction pathway of Ephrin-A5 was corresponding with the activation of Rho and its downstream effector Rho kinase [163]. In the context of commissural axon guidance, nonreceptor spleen tyrosine kinases (Syk) were proposed to act as molecular switch of growth cone attractive and repulsive responses, and ephrin-B3/EphB2 were proposed as candidates in driving Syk-dependent switching at the midline. Unlike Sema3B, which is a secreted factor that possesses longer range ability, ephrin-B3 is expressed at the floor plate and acts with short range activity. These guidance cues might act in different spatial and temporal windows. Thus, proper coordination between attraction and repulsion responses of a complex array of guidance cues plays a crucial role in developing commissural axons [164].

In spite of working independently to mediate chemorepulsion alone, ephrins can form complexes with other types of guidance cues. UNC5c, one subtype of netrin receptors, could form a complex in a ligand-dependent manner with EphB2. This kind of synergistic integration involved SFK signaling, which is a common effector of pathways of both guidance cues [165]. Moreover, a new theory has been proposed in recent years about how ephrin-Eph signaling mediates intercellular communication. It was believed that release of extracellular vesicles, or exosomes, containing Ephs and ephrins might be a part of the intercellular communication in addition of the direct cell contact [166].

Eph/ephrin signaling also plays a role in directing injured axons to reconnect and reestablish their function in the nerve regeneration process. As stated earlier, Schwann cells are required to guide axons across the bridge to the distal stump of the transected nerves. According to Parrinello et al., in the early stages of peripheral nerve repair after transection, through EphB2/ephrin signaling, fibroblasts caused the Schwann cells to migrate out of the nerve stumps to guide regrowing axons across the wound [167]. Later, TGF*β* was further identified as a key mediator of peripheral nerve regeneration after transection where Eph/ephrin signaling was identified as a novel effector of TGF*β*. Their works are further evidences that Schwann cells could interact with regenerating axons thus being important in directional axon guidance, and wound microenvironment is a key determinant of Schwann cell identity [168]. On the other hand, in the model of optic nerve injury of adult mice, EphrinA3 deletion did not affect regeneration, but the absence of EphA4 enhanced the growth of lesioned axons without increasing unwanted axonal branching. These results are likely to be utilized to improve axon regeneration together with other growth-stimulatory treatments of injury in both the CNS and the PNS [169].

#### 3.2.5. Slit and Roundabout

The study on Slit and Roundabout (Robo) was glooming in the 1990s. Slit was identified as a large extracellular matrix protein with four regions containing tandem arrays of a 24-amino-acid leucine-rich repeat with conserved flanking sequences (flank-LRR-flank) and two regions with EGF-like repeats. It played a major role in the development of the specialized midline glial cells and the commissural axon tracts that traverse the midline glia cells [170]. In the olfactory system, Slit acted as a repulsive molecular cue for migrating neurons [171]. Analysis of *slit* mutant phenotype and consequences of transgene expression indicated that Slit acted to repel growth cone away from the ventral midline in *Drosophila* embryos [172].

Large-scale screen for mutations trying to find out the type of the factors that are affecting the axon pathfinding in the CNS in *Drosophila* embryo indicated that mutations in *robo* led to opposite misrouting and caused some of the growth cones to cross the midline that they were not supposed to, suggesting the possible repulsive function of Robo in axon guidance [173]. At this time, it was unknown what midline ligands Robo binds to. Interestingly, the evidences supporting the hypothetical ligand-receptor relationship between Slit and Robo emerged almost at the same time. Two human Robo family genes, two rat Robo family members, and a second Robo-like gene in *Drosophila* were identified. Three Slit homologs in the mouse were identified by in situ hybridization. During embryogenesis, these three *slit* genes and Robo1 were expressed in unique and complementary patterns in the CNS and in other tissues, thereby proposing the ligand-receptor relationship between Slit and Robo [174]. Meanwhile, genetic analysis in *Drosophila* indicated that, in *slit* mutants, growth cones entered the midline but never left it with high expression of Robo level abnormally. Another finding was that *slit* and *robo* displayed dosage-sensitive genetic interactions, suggesting that they might have the same pathway. Thereby, Slit possibly was the midline ligand for Robo [175, 176]. Isolation of vertebrate homologs of the *Drosophila slit* gene verified that Slit protein bound to Robo [177].

Although it had been widely acknowledged that Robo was the receptor of Slit at that time, doubts still existed. For example, as mentioned above, in *slit* mutant mice, the growth cones entered the midline but never left it. But in *robo* mutant mice, growth cones crossed the midline that they do not normally do; the difference probably means that Slit has other receptors. Genetic analysis of *Drosophila* genome revealed that it encoded three Robo families. Result suggested that the *robo*, *robo2* double mutant was largely identical to *slit* mutant (the axons navigated to the midline but did not leave it), meaning that the functions of these two receptors were accounted for all the functions of Slit in midline axon guidance [178]. Then, one year later, the cell surface heparan sulfate (HS) was believed to be involved in the repulsive guidance activities of Slit2 protein, which was important in axon growth and branching of neurons. Previous studies presented evidences regarding binding affinity of HS to Slit2: first, Slit2 protein was found to bind with the column of heparin used for purification. Second, Slit2 protein bound to glypican-1, a member of membrane-associated heparan sulfate proteoglycan, which was completely abolished by heparinase III treatment. In order to evaluate the biological significance of this binding activity between Slit2 and HS, heparinase III was used to remove HS; it turned out that the binding between Slit2 and its receptor Robo-1 was abolished to levels barely detectable by immunofluorescence. On the contrary, with the presence of HS, binding affinity of Robo-1 to Slit2 was enhanced. Furthermore, in the absence of HS, the repulsive activity of Slit2 on the migration of olfactory interneuron precursors was completely abolished. Taken together, these findings demonstrated the important role of HS in repulsive activities of Slit2 [179]. In another experiment, the HS-polymerizing enzyme EXT1 in the embryonic mouse brain was conditionally disrupted, the result showed that the EXT1-null mice displayed severe guidance errors in major commissural tracts, proving the participation of HS in midline guidance [180]. After that, researches have been done to study the role of the carrier proteins of HS in axon guidance, heparan sulfate proteoglycans (HSPGs). HSPGs were presented on the cell surface or in the extracellular matrix, and they were highly negatively charged heterogeneous carbohydrate modifications of some proteins [181]. Two classes of HSPGs were thought to be responsible for carrying HS polymers: the transmembrane Syndecans and the glycosylphosphatidylinositol- (GPI-) linked glypicans. *Drosophila* has one Syndecan gene (*sdc*) and two glypican genes. HSPG *sdc* was proved important in midline axon guidance [182]. The function of HSPG also is related with other types of guidance cues. For example, LON-2/glypican, a kind of HSPG secreted from epidermal cells, was proposed to act as an extracellular modulator of UNC-40/DCC-mediated axon guidance [183].

In mammals, three Slit homologs (Slit1-Slit3) and four members (Robo1-Robo4) of the Robo family were identified. Robo1 and Robo2 are receptors for Slit1-Slit3 with similar binding affinities. In the context of commissural axon guidance, Robo1 and Robo2 were found responsible for some but not all Slit-mediated repulsion, which might imply the existence of another Slit receptor. Robo1 and Robo2 could collaborate to prevent recrossing of postcrossing axons [9]. While Robo1 and Robo2 have some genetic overlap, they have a distinct role in pioneer longitudinal axon trajectories in the CNS. It is suggested that Robo1 acted predominantly to guide pioneer longitudinal axons in ventral tracts and Robo2 in dorsal tract. Additionally, Robo2 has a distinct function in repelling neuron cell bodies from the floor plate [184]. Robo3, which is expressed exclusively in commissural neurons, might possess a complex, central, and multifaceted role in controlling the development of commissural circuits. It is illustrated that, unlike other Robo receptors, Robo3 does not harbor high binding affinity for Slits because of specific substitutions in the first immunoglobulin domain. Robo3 could form complex with DCC, and netrin-1 could bind to DCC and induce Robo3 phosphorylation. Thereby, Robo3 is a component of an attractive netrin-1 receptor mechanism. Specifically, the mutations of the Ig1 domain of mammalian Robo3 might contribute to switch its function. Two splice isoforms of Robo3 with opposite activities were identified: Robo3.1 and Robo3.2. Robo3.1 is expressed in precrossing axons and promotes midline crossing by suppressing the axonal responsiveness to Slit. Robo3.2 localizes to postcrossing axons and contributes to midline crossing [185]. Moreover, Robo3.1A, the longest isoform of Robo3, was suggested that it did not directly bind with Slit on its own but prevent Slit from binding to the surface of cells expressing its close homolog Robo1/2 by downregulating Robo2 protein level [186].

The crosslinks between the Slit/Robo signaling and other guidance cues were presented as well. For example, Robo1 formed complex with DCC and silenced the attractive effect of netrin-1 in growth cones of embryonic *Xenopus* spinal axons [187]. In mammals, netrin-1/DCC-mediated attractive response and Slit/Robo-mediated repulsive response balanced each other and acted together to guide pioneer midbrain longitudinal axons [188]. Lately, a crosslink between Slit/Robo and semaphorin/plexin signaling in commissural axon guidance was found. The N-terminal fragment on Slit specifically bound with plexinA1 and was independent of Robos and neuropilins, indicating that plexinA1 is a new Slit receptor that mediates both semaphorin and Slit activities of repulsive response during commissural axon guidance [189].

#### 3.2.6. Morphogens as Nonconventional Guidance Cues

Morphogens are signaling molecules produced in certain regions but can form a long-range gradient from their source. Three types of morphogens have been regarded as nonconventional guidance cues: Wingless/Int-1 (Wnt), sonic hedgehog (Shh), and bone morphogenetic protein (BMP) families; all of them are critical in organizing body patterns [190].

*(1) Wingless/Int-1 (Wnt)*. Wnt proteins are a family of morphogens that have been shown to function as axon guidance cues and were studied most in anterior-posterior (A/P) guidance of commissural axons in the spinal cord or in the bilateral symmetrical nervous system. In particular, Wnt proteins are expressed in a decreasing anterior-posterior gradient in the floor plate. Wnt proteins are coupled to various receptors and then activate different downstream pathways, which can be categorized into canonical (*β*-catenin dependent) and noncatenin (*β*-catenin independent) signaling pathways. Noncanonical Wnt/*β*-catenin signaling was shown to regulate axon guidance in the developing nervous system where Wnts bind to several cell membrane receptors: a class of seven-transmembrane protein receptors called Frizzled, as well as the Ryk/Derailed receptor tyrosine kinase. In *C. elegans* genome, five Wnts (EGL-20, CWN-1, CWN-2, LIN-44, and MOM-2) and four Frizzled receptors (MIG-1, CFZ-2, LIN-17, and MOM-5) were identified, and all of them were suggested to participate the pathfinding process of a pair of bilaterally symmetric motor neurons [191]. In the *Drosophila* embryonic nervous system, Derailed, an atypical receptor tyrosine kinase expressed on axons projecting in the anterior commissure, was identified as a receptor of Wnt5 [192]. In *C. elegans*, *Drosophila*, and mouse, Ryk (receptor related to tyrosine kinase) serve as a Wnt receptor [193]. Furthermore, in the rat embryonic nervous system, Wnt proteins acted as axonal attractants in midline axon guidance with the receptor Frizzled3 expressed in the spinal cord. This Wnt/Frizzled pathway controlled the anterior turning of the spinal cord commissural axons after midline crossing. However, this attractive response was thought to be independent of Ryk/Derailed signaling[194]. While in zebrafish, muscle-specific receptor tyrosine kinase unplugged/MuSK bound with Wnt11r in muscle fibers to restrict growth cone guidance, where MuSK is a muscle-specific receptor tyrosine kinase [195]. Later research proposed the important role of Wnt/Ryk calcium signaling mechanisms in regulating repulsion response in cortical cultures. When axons sensed guidance cues, calcium was released from internal stores and entered through TRPC channels, which process was an important source of calcium. And calcium/CaMKII (calcium/calmodulin-dependent protein kinase II) was proposed as a downstream effector of Wnt/Ryk signaling [196].

The separate class of Wnt pathways, canonical *β*-catenin-dependent Wnt signaling pathway, was shown to play a role in axon regeneration. Several studies suggested that the canonical *β*-catenin-dependent Wnt signaling pathway is activated after SCI and optic nerve injury and could promote functional recovery [197–200]. While the specific role and mechanism of it in guiding regrowth axons remain to be elucidated, Wnt expression after nerve injury may have therapeutic potential in promoting axon regeneration and functional recovery.

Latest researches built the links between Wnts and growth cone cytoskeleton. Between calcium signaling and the reorganization of dynamic microtubules lays tau. Tau is phosphorylated at the Ser 262 microtubule-binding site by CaMKII which was required for Wnt5a-induced axon repulsive turning [201]. Another crosslink was related to the planar cell polarity (PCP) signaling pathway, a signaling complex consisted of GIT1/PIX/Rac/PAK. In zebrafish, the so-called commissural primary ascending (CoPA) is the earliest born spinal commissural neuron to navigate the midline and turn ipsilaterally. In the mutant model, the PCP signaling pathway was suggested to modulate the anterior guidance decisions of CoPA axons [202]. This complex was believed to control growth cone motility as well [203]. The PCP signaling pathway was important in commissural axons to turn anteriorly in a Wnt gradient after midline crossing, where Wnt5a increased Frizzled3 endocytosis, which was correlated with filopodia elongation. And then Frizzled3 was recycled [204]. Taken together, Wnts, coupled with their receptors, play a role in axon pathfinding process.

*(2) Sonic Hedgehog (Shh)*. Shh is secreted by floor plate in the spinal cord. It patterns the ventral spinal cord in vertebrates and promotes ventral migration of commissural axons. Boc served as receptor of Shh in mediating attractive response of commissural axons in mice embryo [205]. It is worthwhile mentioning that the function of Shh and its receptors in the establishment of binocular vision in vertebrates is much clearer. In higher vertebrates, visual formation is relayed to the brain once retina has received information. Visual information is delivered through retinal ganglion cell (RGC) neurons, whose axons extend towards the midline and meet at the so-called optic chiasm at the midline. Then, each RGC axon has to decide to either cross or turn away from the chiasm midline and grow towards the brain. As RGC axons need to project either contralaterally or ipsilaterally and continuously to the brain to enable binocular vision, the optical chiasm is regarded as a choice point during development. In mice, two major types of RGC populations exist: (1) the Islet2-expressing contralateral projecting (c)RGCs, which both produce and respond to Shh and (2) the Zic2-expressing ipsilateral projecting RGCs (iRGCs), which lack Shh expression [206]. In Fabre et al.'s study, Boc was found enriched in ipsilateral RGCs in the developing retina of mice embryo and that Shh repelled ipsilateral RGC axons at the optic chiasm via Boc [207]. Later, this process was elucidated further. It is proposed that despite the fact that ipsilateral RGCs were repelled away from the midline by Shh, the mRNA for Shh was not found in optic chiasm. Instead, Shh protein was produced by contralateral RGCs, transported anterogradely along the axon, and accumulated at the optic chiasm to repel ipsilateral RGC axons. Furthermore, this study established that Shh can serve as a diffusible cue and can act at axon guidance midline choice points [208]. Shh also mediated olfactory sensory neuron axons to enter their target glomeruli and to branch in the target region in rat embryos [209].

Another receptor of Shh is Ptc. Upon binding Shh, Ptc released Smoothened (Smo), a transmembrane protein, which then mediated Shh signaling by activating intracellular molecules including the Gli family transcription factors [210]. The signaling mediator Smo was important for normal projection of commissural axons to the floor plate. Shh was suggested to collaborate with netrin-1, both served as chemoattractant for commissural axons in mouse embryos while Shh acted via Smo [211]. Moreover, it is proposed that Shh guided commissural axons via a rapidly acting in a transcriptionally independent manner but activated SFKs in a Smo-dependent manner and caused changes in the growth cone skeleton, where graded SFK activity could mediate axon turning [212].

Recent research elucidated that in Shh-mediate commissural axon repulsion, heparan sulfate proteoglycan glypican1 (GPC1) functioned together with hedgehog-interacting protein [213]. And as previously stated in Wnt, PCP signaling also plays a role in Shh/Smo-mediated response in the context of commissural axon guidance in rodents. It is proposed that Shh in the ventral midline could switch on Wnt/PCP signaling by inhibiting the mRNA level of Shisa2 in the cell body. In turn, the inhibition allowed Frizzled3 to be trafficked to the cell surface, resulting in the activation of Wnt/PCP signaling in commissural axon growth cones. Presumably, the link between Shh and PCP signaling may also occur in other developmental processes. Taken together, it is suggested that the switch mechanism is highly sophisticated to ensure proper changes of responsiveness for axons at intermediate targets [214]. Just like other guidance cues, Shh is associated with changes in the growth cone cytoskeleton as well. According to Lepelletier et al., the turning of rat commissural axons up a Shh gradient needed *β*-actin protein synthesis at the growth cone, and Shh-induced local translation of *β*-actin required zipcode-binding protein 1 (ZBP1), which is an mRNA binding protein that transport *β*-actin mRNA and releases it for local translation upon phosphorylation. Meanwhile, ZBP1 activity was required for correct commissural axon guidance *in vivo.* These results identified a new mediator, ZBP-1, for noncanonical Shh signaling in axon guidance [215].

*(3) Bone Morphogenetic Protein (BMP) Family*. During neural tube development, several members of the BMP family are expressed in the dorsal roof plate (RF), such as BMP7, growth/differentiation factor 7 (GDF7), and BMP6. It is believed that their main function is to control the induction and differentiation of dorsal interneurons. BMPs exerted their function as a heterodimer of BMP7 and GDF7. Early in the 1990s, BMP7 was found to act as roof plate-derived chemorepellent that repelled the commissural axons away from the dorsal root in the developing spinal cord *in vitro* [216]. Experiment on gene mutant mice suggested that GDF7 enhanced the axon-orienting activity of BMP7 as chemorepellent for commissural axons [217]. In cultured embryonic *Xenopus laevis* spinal neurons, BMP7 gradient was suggested to attract and repel growth cone in a time-dependent manner through regulating ADF/cofilin with different signaling pathways [218]. The canonical BMP receptor (BMPR) complex consisted of type I and II serine/threonine kinases. BMPRIA and BMPRIB are two subtypes of type I BMPR. However, it seemed that BMPRIB was necessary and sufficient in mediating commissural axon guidance in chicken embryos, as well as in reorientation in mouse embryos [219]. Another experiment studied the function of BMPRIB on RGC. BMP receptors IA, IB, and II were expressed in the developing retina, while BMPRIB was expressed exclusively in the ventral retina during embryonic development and was required for normal ventral ganglion cell axon to target the optic nerve head [220]. A neural-specific secreted antagonist of BMP signaling was identified: brorin, expressed in human, mice, and zebrafish. In zebrafish, *brorin* gene was primarily expressed in developing neural tissues. Result suggested that *brorin* was essential for the appropriate expression of axon guidance molecules [221]. A recent study proposed the involvement of a kind of Na^+^/Ca^2+^ exchanger proteins, NCX-9, in axon guidance in *C. elegans*. NCX-9 secreted guidance cue UNC-129/BMP to control the left/right patterning in neural circuit formation [222].

BMPs are also important in regrowing axons in adults after injury. Setoguchi et al.'s experiment suggested that taking advantage of gene modifications which could attenuate BMP signaling promoted functional recovery after SCI of mice [223]. Another study found that the expression of BMP2/4 witnessed an increase in oligodendrocytes and astrocytes around the injury site following the spinal cord contusion, while the application of BMP antagonist led to promoted regrowth of the corticospinal tract and enhanced locomotor activity. Therefore, BMPs may play a role in inhibiting axonal regeneration and limiting functional recovery following injury to the CNS [224]. However, BMP-Smad signaling might possess a positive role in axon regeneration. A so-called conditioning lesion model revealed the function of the transcription factor Smad1. Neuronal Smad1 was upregulated and phosphorylated Smad1 accumulated in the nucleus after axotomy of the peripheral branch of adult dorsal root ganglia, suggesting that continued presence of Smad1 was required to maintain the growth program [225]. It is worthwhile mentioning that Smad1 is developmentally regulated; reactivated Smad1 signaling in adult dorsal root ganglia resulted in rekindling of axon growth potential. Taken together, BMP/Smad could be a therapeutic target to promote axon regeneration after nerve injury [226].

## 4. Signaling Pathways

**Figure 4:**
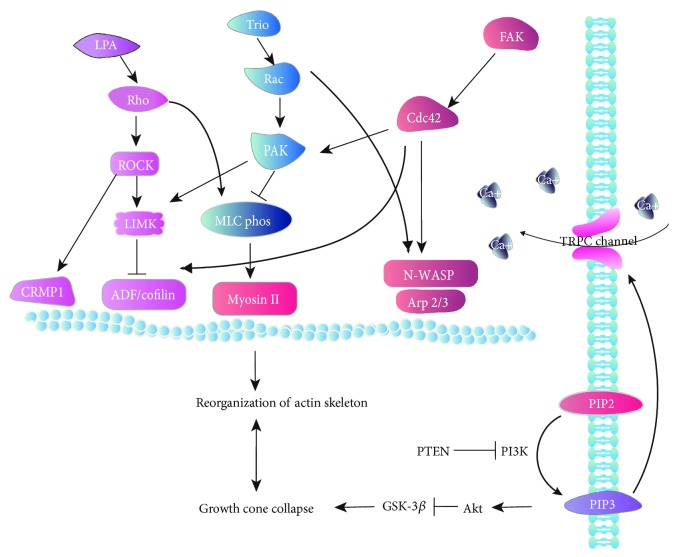
Summarization of some signaling pathways in mediating the response of growth cone towards multiple molecular cues.

The key to understand the mechanisms of axon guidance is to link the regulation of growth cone cytoskeleton to the reception of guidance cues. Here, we summarize some of the signaling pathways in terms of the category of the guidance cues (Table 1) and the network of some signaling pathways (Figure 4).

### 4.1. GTPase-Related Pathways

The role of GTPase in axon guidance is becoming more evident [228, 244]. The Rho family GTPases are mainly responsible for rearrangement of F-actin skeleton. The activities of Rho GTPases are regulated by two classes of proteins: RhoGAPs and RhoGEFs. Those GTPases are inactivated to guanosine diphosphate (GDP) by hydrolysis and regulated by GTPase-activating proteins (GAPs). In contrast, guanine nucleotide exchange factors (GEFs) promote the release of bound GDP and allow for binding to GTP. Briefly speaking, RhoGEFs switch GTPases on, while RhoGAPs switch them off [245, 246]. GTPases interact with the so-called effector proteins in the downstream that can mediate the effect of them. Three major small GTPases are important in regulating guidance: Rho, Rac, and Cdc42.

ROCKs (also known as Rho kinases), a class of serine/threonine kinases, were the first downstream effectors of Rho to be discovered. Two main classes of the ROCK family: ROCK-1 and ROCK-2, were identified. Normally, the RhoA/ROCK pathway was thought to inhibit axon growth [247, 248], since the inhibition of ROCK activity promoted axon growth and regeneration [249, 250]. Blockage of ROCK by cell-permeable inhibitor Y27632 led to axon misguidance, together with many other studies that suggested the involvement of the RhoA/ROCK pathway in axon guidance [242, 251, 252].

A classic study revealed the link between Rho and actin cytoskeleton: LIM-kinase (LIMK) was phosphorylated and activated by ROCK, in turn, LIMK phosphorylated cofilin, which is a kind of actin-depolymerizing factor and is essential for turnover of actin filaments. Phosphorylated cofilin resulted in decreased depolymerization of F-actin [253, 254]. Later study proposed that actin depolymerization factor (ADF), which is mentioned above, was regulated by LIMK as well. LIMK phosphorylated and inactivated ADF/cofilin, resulted in net increase in the cellular filamentous actin. Recent study proposed that active cofilin 1 was able to restore the response of injured axons to attractive cues, and it might be a potential target that can be utilized to reinstate the regeneration capacity of axons in neurodegenerative diseases [255]. In spite of ADF/cofilin in this RhoA/ROCK pathway, the myosin light chain (MLC) kinase phosphorylation was found pivotal as well. In mammalian cells, nonmuscle myosins are regulated by MLC on Ser19 by the myosin light chain kinase (MLCK). MLCK-mediated phosphorylation of MLC regulated actin-myosin II interactions in cytoskeleton dynamics [256]. In an experiment studying the dendritic cells trafficking, during entry into lymphatics, Sema3A was found to induce actomyosin contraction by activating MLC phosphorylation; this effect could be attenuated by blockage of ROCK activity [230]. Another study was consistent with this result that activated ROCK increased the phosphorylation rate of MLC thereby causing neurite retraction [257]. In these studies, lysophosphatidic acid (LPA) was important that could both induce growth cone collapse and neurite retraction. LPA-induced growth cone collapse and LPA-induced neurite retraction were both mediated by activating ROCK [257, 258]. Another important family involved in the Rho signaling pathway is the collapsin response mediator protein (CRMP) family. It was phosphorylated by ROCK in response to LPA, this signaling pathway was related with semaphorins' activity [228]. Then, CRMP1, a member of the CRMP family, was proposed to interact with Speedy A1 (Spy1), a member of the Speedy/RINGO family. Spy1 interrupted the binding process of CPMR1 with actin and mediated Sema3A-induced growth cone collapse in the regeneration process after sciatic nerve crush injury [259].

Rac is also important. GEF Trio was essential in photoreceptor axon guidance in *Drosophila*. The two Trio GEF domains activated Rac1, which in turn, activated p21-activated kinase (PAK) that regulated actin dynamics, thereby controlling the direction of growth cone [233]. Rac1 also activated LIMK and triggered phosphorylation of cofilin [260]. The crosslink between Rho and Rac is MLCK, which also served as a downstream effector for PAK. Cellular effects of PAK were indicated to be mediated through the phosphorylation and inactivation of MLCK and a decrease in MLC phosphorylation, thus reduced activity of myosin II [256, 261].

Cdc42 responses to both attractive and repulsion molecular cues and causes change in growth cone motility. This signaling was modulated by focal adhesion kinase (FAK) activity, which served as a common upstream modulator for BDNF and Slit, whereas BDNF activated Cdc42 while Slit2 inactivated Cdc42 [235, 262]. An important downstream effector of Cdc42 is N-WASP (neural Wiskott-Aldrich syndrome protein) and the Arp2/3 complex. Arp2/3 is a kind of actin-binding protein and was implicated to be a regulator of actin dynamics in nonneuron cells [263], while its role in growth cone guidance has been unclear and controversial yet. Opinions diverse on whether it is necessary in filopodia formation in the growth cone [264]. Latest research indicated that the role of Arp2/3 on growth cone motility was crucial for guidance regarding L1, but not on laminin, that the function of Arp2/3 depended on the substrate [265]. Cdc42 activated N-WASP, which continued to activate Arp2/3 and caused actin assembly [266, 267]. In addition, Cdc42 has crosslinks with other small GTPases. In BDNF-mediated change in filopodial dynamics of the growth cone, Cdc42 mimicked the activation of ADF/cofilin that resulted from BDNF treatment. The interaction between these two small GTPases occurred upstream of ROCK [81]. Other crosslinks lay between Cdc42 and Rac1 are Pak and N-WASP. Research suggested that Rac1 was an activator of N-WASP and even more potent than Cdc42 [268]. Also, Pak1 bound both Cdc42 and Rac1 and led to activation of LIMK. Thus, these activated GTPases regulate actin depolymerization through Pak and LIMK [269].

### 4.2. Phosphoinositides: PI3K/Akt Pathway

The PI3K/Akt pathway is very important in regulating tumor cell proliferation, growth, survival, and angiogenesis as well as in promoting axon outgrowth in both the CNS and the PNS, serving as the survival pathway downstream of neuronal growth factors [270]. Previous studies suggested that activity of the second messenger phosphatidylinositol 3, 4, 5-trisphophate 3 (PIP3) and Akt (also called protein kinase B) mediated growth cone chemoattraction. PI (4,5) P2 can be phosphorylated to PIP3 by phosphatidylinositol 3 kinase (PI3K). PIP3 elevation resulted in increased activity of its downstream regulator Akt and activated the activity of TRPC, which was indicated to mediate BDNF and netrin-1 signaling. However, with suppressed activity of phosphatase and tensin homolog (PTEN), which is an endogenous inhibitor for the PI3K/Akt pathway, chemorepulsion was blocked with negative cues. The result suggested that activation of this PI3K/Akt signaling pathway regulated chemoattractive response towards growth cone [271, 272]. Furthermore, it is shown that PTEN negatively regulated PIP3 levels of the growth cone and mediated chemorepulsion but not chemoattraction [272]. Latest researches proposed a role for PTEN in inhibiting axon regeneration and functional recovery after spinal cord injury [273, 274]. Downstream effector of Akt: glycogen synthase kinase (GSK)-3*β*, is involved in axon guidance as well. Activation of GSK-3*β* induced growth cone collapse, while the process could be blocked by Akt-activated phosphorylation of GSK-3*β* [229, 232]. Latest research demonstrated that in the mature nervous system, peripheral axotomy activated PI3K/GSK3 signaling, and this signaling was conveyed by the induction of Smad1. Together, PI3K/GSK3/Smad1 could promote sensory axon regeneration, but the extent of this signaling pathway contributes to axon pathfinding process in regeneration, and subsequent functional recovery remains to be determined [275].

## 5. Final Thoughts

Growth cone motility is of fundamental importance to its turning ability which allows it to sense the attractive and repulsive cues surrounding it and make directional decision. Several well-identified guidance cues and other cell adhesion molecules that could mediate axon guidance keep exerting their effects on the growth cone, whether by forming concentration gradient or acting locally, and continuously influence actin cytoskeleton and microtubules stability and dynamics of the growth cone [276]. Regulation of growth cone motility is applied to both developing and regenerating axons that the guidance cues could regulate growth cone motility and substantially, its turning ability of navigation in both processes where multiple signaling pathways bridge the effects of guidance cues to the growth cone to mediate axon guidance.

The effects of guidance cues was studied to the most details in the developing nervous system, but not in the regeneration process. Although some of the guidance cues were proved to enhance axon outgrowth, and some were thought to improve functional recovery, such as locomotor recovery, the exact roles and mechanisms of these guidance cues in axon guidance in the regeneration process are still not clear. Factors influencing the regeneration process are complex, for example, the injury site and the size of injured nerve. Despite surgical nerve repair and other strategies used clinically to promote regeneration, function recovery is usually compromised especially in the cases of spinal cord injury due to the lost capability of neurons in the CNS to regenerate. In peripheral nerve injury, motor, sensory, and autonomic functions might loss. Poor functional recovery in part comes down to misdirection since regrowing axons fail to reinnervate target organs and thus having negative impact on functional outcomes. In such cases, axon guidance needs to be precise in the navigation process. Notably, nerve fibers could regenerate without navigating appropriately to its original targets. Since that functional recovery is the most tightly relevant outcome to successful peripheral nerve regeneration, it is rational to illustrate whether the nerves make successful end organ connections by functional analysis [277].

On the other hand, it is widely accepted that the motility of growth cone is attributed to its actin and microtubule cytoskeleton and that affluent signaling pathways weave a subtle network to connect molecular cues with the cytoskeleton. However, these underlying mechanisms in axon guidance also apply to other processes, such as cell proliferation, cell migration, cell adhesion, and neurodegenerative diseases. It would be intriguing to know what the influences are in terms of the interplays among axon guidance and other processes in integrated scenario. Future orientation might be related with electric stimulation remedy. It is suggested that electroacupuncture promoted nerve regeneration in both the CNS and the PNS in rodents. Induction secretion of some guidance cues (e.g., endogenous NT-3) and neurotrophic factors were observed when applying electric stimulation [278, 279]. However, less has been known about whether electric stimulation can regulate axon guidance. Since varies guidance cues are shared by nerve regeneration and axon guidance, it would be worthy examing and comparing the rate of misdirection after nerve injury with or without electric stimulation, thereby exploring the function of electric stimulation in axon guidance. Further studies will help us have a better understanding of the intrinsic mechanisms of axon pathfinding process in both the developing nervous system and the nerve regeneration process and hopefully to provide foundation for clinic treatment.

## Figures and Tables

**Table 1:** Suggested signaling pathways of guidance cues.

Target		Suggested mechanisms
Semaphorins	Sema 3A	Rac1 amino acids 17-13 [134], LIMK/cofilin [227], CRMP family [228], GSK-3*β* [229], RhoA/ROCK [230], ERM family [135], Rab family [136]
	Sema 3F	PI3K/Akt, MEK/ERK [231]
	Sema 4D	GSK-3*β* [232]

Slit		Dock, Pak, the Rac1/Rac2/Mtl small GTPases [233], Fyn, Cdk5 [234], FAK/Cdc42 [235]

Ephrins	Ephrin-B2	Nck/Pak signaling complex [236]
	Ephrin-A	RhoA activation, Cdc42 and Rac1 inhibition [237], Rho/ROCK [163], Ret [91], Tsc2-Rheb [238], RacGAP *α*2-chimaerin [161]

Netrin	Netrin-1	Trio, Rac1 [239], cAMP [100], JNK-1 [126], TRPC channel [240]

Neurotrophic factors	NGF	TrkA receptor [65], cAMP [241], ROCK [242], PI3K, PLC-*γ* [243]
	BDNF	TRPC channel [82], ADF/cofilin (AC) [67], PI3K, PLC-*γ* [243], FAK/Cdc42 [235]
	GDNF	Ret [91]

Nonconventional guidance cues	Wnt	Wnt/Frizzled [194], PCP [204], Ryk/Derailed [192, 193]
	Shh	SFKs [212]
	BMP	LIMK, ADF/cofilins [218]

Abbreviations: LIMK: LIM kinase, PI3K: phosphatidylinositol 3 kinase, PLC-*γ*: phospholipase C-*γ*, MEK: mitogen-activated protein kinase, ERK: extracellular signal-regulated kinase, ROCK: RhoA kinase, TRPC: transient receptor potential canonical, ERM: ezrin/radixin/moesin, FAK: focal adhesion kinase, PCP: planar cell polarity, SFKs: Src family kinases.
